# Carriers of the Complex Allele *HFE* c.[187C>G;340+4T>C] Have Increased Risk of Iron Overload in São Miguel Island Population (Azores, Portugal)

**DOI:** 10.1371/journal.pone.0140228

**Published:** 2015-10-26

**Authors:** Claudia C. Branco, Cidália T. Gomes, Laura De Fez, Sara Bulhões, Maria José Brilhante, Tânia Pereirinha, Rita Cabral, Ana Catarina Rego, Cristina Fraga, António G. Miguel, Gracinda Brasil, Paula Macedo, Luisa Mota-Vieira

**Affiliations:** 1 Molecular Genetics and Pathology Unit, Hospital of Divino Espirito Santo of Ponta Delgada, EPE, São Miguel Island, Azores, Portugal; 2 Instituto Gulbenkian de Ciência, Oeiras, Portugal; 3 BioISI – Biosystems & Integrative Sciences Institute, Faculty of Sciences, University of Lisboa, Lisboa, Portugal; 4 Gastroenterology Department, Hospital of Divino Espirito Santo of Ponta Delgada, EPE, São Miguel Island, Azores, Portugal; 5 Hematology Department, Hospital of Divino Espirito Santo of Ponta Delgada, EPE, São Miguel Island, Azores, Portugal; 6 Pneumology Department, Hospital of Divino Espirito Santo of Ponta Delgada, EPE, São Miguel Island, Azores, Portugal; 7 Internal Medicine Department, Hospital of Divino Espirito Santo of Ponta Delgada, EPE, São Miguel Island, Azores, Portugal; Hospital Israelita Albert Einstein, BRAZIL

## Abstract

Iron overload is associated with acquired and genetic conditions, the most common being hereditary hemochromatosis (HH) type-I, caused by *HFE* mutations. Here, we conducted a hospital-based case-control study of 41 patients from the São Miguel Island (Azores, Portugal), six belonging to a family with HH type-I pseudodominant inheritance, and 35 unrelated individuals fulfilling the biochemical criteria of iron overload compatible with HH type-I. For this purpose, we analyzed the most common *HFE* mutations– c.845G>A [p.Cys282Tyr], c.187C>G [p.His63Asp], and c.193A>T [p.Ser65Cys]. Results revealed that the family’s HH pseudodominant pattern is due to consanguineous marriage of *HFE*-c.845G>A carriers, and to marriage with a genetically unrelated spouse that is a -c.187G carrier. Regarding unrelated patients, six were homozygous for c.845A, and three were c.845A/c.187G compound heterozygous. We then performed sequencing of *HFE* exons 2, 4, 5 and their intron-flanking regions. No other mutations were observed, but we identified the -c.340+4C [IVS2+4C] splice variant in 26 (74.3%) patients. Functionally, the c.340+4C may generate alternative splicing by *HFE* exon 2 skipping and consequently, a protein missing the α1-domain essential for HFE/ transferrin receptor-1 interactions. Finally, we investigated *HFE* mutations configuration with iron overload by determining haplotypes and genotypic profiles. Results evidenced that carriers of *HFE*-c.187G allele also carry -c.340+4C, suggesting in-*cis* configuration. This data is corroborated by the association analysis where carriers of the complex allele *HFE-*c.[187C>G;340+4T>C] have an increased iron overload risk (RR = 2.08, 95% CI = 1.40−2.94, p<0.001). Therefore, homozygous for this complex allele are at risk of having iron overload because they will produce two altered proteins—the p.63Asp [c.187G], and the protein lacking 88 amino acids encoded by exon 2. In summary, we provide evidence that the complex allele *HFE*-c.[187C>G;340+4T>C] has a role, as genetic predisposition factor, on iron overload in the São Miguel population. Independent replication studies in other populations are needed to confirm this association.

## Introduction

Iron overload in humans is associated with a variety of acquired and genetic conditions, the most common being the hereditary hemochromatosis type-I (HH, OMIM #235200), an autosomal recessive disorder caused by mutations in the *HFE* (*High Iron Fe*, OMIM *613609) gene [1]. *HFE* encodes an *HLA*-A class 1-like protein and is located on 6p21.3, 4 megabases (Mb) telomeric to the human leukocyte antigen region (*HLA*).

Two *HFE* mutations– c.845G>A [p.Cys282Tyr] and c.187C>G [p.His63Asp]–were originally described in association with HH [2]. The majority (60% to 90%) of clinically diagnosed probands were homozygous for c.845A [p.282Tyr], and 5% were c.845A/c.187G compound heterozygous. In terms of molecular pathology, the c.845A is the most severe mutation. Its frequency decreases from the north to the south of Europe and is very low in non-European derived populations, such as Asians [3], Africans [4] and Ashkenazi Jewish [5]. The second originally described mutation– c.187G [p.63Asp]–is found as a highly frequent polymorphism in general populations [2]. Nonetheless, it was observed at an increased frequency in HH patients’ chromosomes that do not carry the c.845A mutation [2,6], suggesting a possible role as a modifier of iron overload. A third sequence variant in the *HFE* gene − c.193A>T [p.Ser65Cys] − was increased in some HH patients’ groups in comparison to healthy controls [7,8]. Despite the fact that individuals with the c.845A/c.193T genotype may have an increased risk to express a milder form of HH, the penetrance of this genotype is low and other genetic and environmental factors may influence the expression of iron overloading [9]. Other *HFE* single nucleotide polymorphisms and rare variants have been implicated in hemochromatosis [10,11], including intronic splicing mutations, such as c.340+4T>C [IVS2+4T>C] [12] and c.1008+1G>A [IVS5+1G>A] [13]. For example, Floreani and colleagues [11] showed that two *cis*-variants– c.193T and c.340+4C –generate alternative splicing by *HFE* exon 2 skipping. The corresponding protein misses the α1-domain, which is essential for the interaction of HFE with TfR1 (Transferrin Receptor-1) [14].

Despite recent advances, a better understanding of the molecular basis of iron overload is needed in order to improve patients' disease outcome through early diagnosis and treatment. Here, we conducted a hospital-based case-control study of individuals living in the Azorean island of São Miguel in order to genetically characterise a family with pseudodominant inheritance of hereditary hemochromatosis (HH) type-I, and an additional group of unrelated patients that fulfilled the biochemical criteria of iron overload compatible with HH type-I. To that end, we genotyped four *HFE* mutations/variants– c.845G>A [p.Cys282Tyr], c.187C>G [p.His63Asp], c.193A>T [p.Ser65Cys], and c.340+4T>C [IVS2+4T>C]–and performed an association analysis of *HFE* haplotypes and genotypic profiles with this condition. Since several *HLA* haplotypes have been associated with *HFE-*c.845A and -c.187G, we also studied the *HLA*-A and -B group alleles and haplotypes linked to these mutations in the São Miguel Island.

## Material and Methods

### Ethics statement

The present investigation follows the international ethical guidelines and was approved by the Health Ethics Committee of the Hospital of Divino Espirito Santo of Ponta Delgada, EPE (HDES). The study design includes, from all participants, written informed consent, confidentiality, and an abandonment option in the case of expressed will.

The general population consists of 469 DNA samples of unrelated healthy blood donors from São Miguel Island selected from the anonymized Azorean DNA bank, located at HDES. This DNA bank was established after approval by the Health Ethics Committee and follows, as well, the international ethical guidelines for sample collection, processing, and storage.

### São Miguel Island population (Azores): Bio-demographic data and general population sampling

São Miguel is the largest (747 Km^2^, measuring 64 km from east to west and 8 to 15 km wide) and the most populated Azorean island (137,699 inhabitants; 55.9% total Azores population; Portugal Census, 2011). This island was uninhabited when discovered in 1427. Nowadays, the island is divided in six municipalities: Ponta Delgada (PDL, 68,809 inhabitants, 49.9%), Ribeira Grande (RG, 32,112 inhabitants, 23.3%), Lagoa (LAG, 14,442 inhabitants, 10.5%), Vila Franca do Campo (VFC, 11,229 inhabitants, 8.1%), Povoação (POV, 6327 inhabitants, 4.6%) and Nordeste (NOR, 4937 inhabitants; 3.6%). Around half of the São Miguel population lives in small rural localities. The rural area is characterized by agriculture and cattle-breeding economy, and its inhabitants show great similarity in life style, as well as in eating habits. Regarding iron metabolism, the literature review did not reveal any previous study on iron overload in this population.

The general population cohort is composed of 469 DNA samples of unrelated healthy blood donors from São Miguel Island (Azores, Portugal). This sample was geographically representative of the six municipalities of the island: PDL (n = 176), VFC (n = 87), RG (n = 76), POV (n = 51), NOR (n = 41) and LAG (n = 38). Ninety-seven percent of the subjects studied have parents born in the same locality.

### Iron overload patients

A total of 41 patients (Table 1) were clinically characterized by six physicians (Internal Medicine, Gastroenterology, Hematology, and Pneumology departments) at the HDES. Six patients belong to one family studied in the context of a family screening of hemochromatosis. They live in a small rural locality with less than 600 inhabitants. The remaining 35 unrelated patients were referred for *HFE* genotyping, since they fulfilled the biochemical criteria of iron overload compatible with the classical form of hereditary hemochromatosis (type-I)– 1. serum ferritin > 400 ng/mL (males) or > 300 ng/mL (females), and/or 2. transferrin saturation (TS) > 50% (males) or > 45% (females), and/or 3. serum iron > 160 μg/dL (males) or > 145 μg/dL (females). Patients with evidence of secondary iron overload, namely exogenous iron intake, hepatitis B or C infection, and daily alcohol consumption higher than 60g, were excluded from the study. All patients and their parents were born in São Miguel Island, being the majority with both parents born in the same municipality (66%; Table 1). Blood samples were collected by venipuncture into dry and EDTA-K_3_ tubes for biochemical and mutation analysis, respectively. Genomic DNA was extracted using the PUREGENE^®^ DNA Purification (Gentra systems Inc.) or Citogene^®^ DNA Purification (Citomed) kits. Serum transferrin, iron, and transaminases (aspartate transaminase, AST, and alanine transaminase, ALT) were measured on a Roche/Hitachi 912 by an immunoturbidimetric, colorimetric assays, and UV test, respectively. Transferrin saturation was calculated according to the formula (Fe/ (transferrin×1.4)) x 100. Serum ferritin was determined using the AxSYM Ferritin Assay (Abbott Laboratories).

**Table 1:** Demographic, clinical, biochemical and genetic data from the 41 iron overload patients from São Miguel Island.

Demographic data			Clinical manifestations							Biochemical tests					Genetic data					
															*HFE* genotype				*HLA* genotype	
ID	Sex	Father/mother municipalities	Age at presentation (years)	Hyperpigmentation	Type I Diabetes	Cardiopathy	Arthropathy	Hepatic cirrhosis	Hepatocellular carcinoma	Serum ferritin (ng/mL)	TS (%)	Serum iron (μg/dL)	AST (U/L)	ALT (U/L)	c.845G>A	c.187C>G	c.193A>T	c.340 +4 T>C	A	B
**Family patients affected with hereditary hemochromatosis (pseudodominant transmition)**																				
III.3	M	NOR/RG	74	No	No	No	Yes	No	No	426	50^b^	155^b^	ND	ND	**AA**	CC	AA	TT	02, 02	44, 55
IV.7^†^	M	NOR/NOR^a^	42	Yes	No	Yes	Yes	Yes	No	>1000	87	183	64	61	**AA**	CC	AA	TT	01, 02	35, 55
IV.8^†^	M	NOR/NOR^a^	40	Yes	No	Yes	Yes	Yes	No	>1000	80	212	ND	ND	**AA**	CC	AA	TT	01, 02	35, 55
IV.12	F	NOR/NOR^a^	37	No	No	No	No	No	No	165^c^	55	108^b^	17	16	**AA**	CC	AA	TT	01, 02	35, 55
V.10	F	NOR/RG	20	No	No	No	No	No	No	52^c^	58	228	21	12	G**A**	C**G**	AA	T**C**	02, 24	15, 55
V.11	M	NOR/RG	18	No	No	No	No	No	No	856	78	338	29	17	G**A**	C**G**	AA	T**C**	02, 24	49, 55
**Unrelated patients with biochemical evidence of iron overload**																				
1	F	NOR/NOR^a^	69	Yes	No	No	Yes	No	No	541	114	294	75	50	**AA**	CC	AA	TT	01, 24	14, 18
2	M	VFC/VFC	46	Yes	No	No	Yes	No	No	582	46^b^	128^b^	23	26	**AA**	CC	AA	TT	03, 33	14, 27
3	M	VFC/VFC^a^	43	Yes	No	No	Yes	Yes	No	>2000	75	242	181	180	**AA**	CC	AA	TT	03, 03	27, 50
4	M	PDL/POV	29	No	No	No	No	No	No	895	100	223	ND	ND	**AA**	CC	AA	TT	01, 29	35, 38
5†	M	PDL/PDL^a^	51	Yes	Yes	Yes	Yes	Yes	No	>1000	93	182	99	74	**AA**	CC	AA	TT	24, 24	15, 15
6	M	PDL/PDL	50	No	No	No	No	No	No	>2000	108	199	31	64	**AA**	CC	AA	TT	02, 33	14, 44
7	F	PDL/PDL^a^	48	No	No	No	No	No	No	447	37^b^	94^b^	28	43	G**A**	C**G**	AA	T**C**	01, 03	44, 58
8	M	PDL/PDL	20	No	No	No	No	No	No	585	44^b^	122^b^	83	176	G**A**	C**G**	AA	T**C**	03, 23	18, 35
9	M	ND	50	Yes	Yes	No	No	Yes	No	>2000	106	66^b^	107	61	G**A**	C**G**	AA	T**C**	29, 29	44, 45
10	F	RG/RG^a^	51	No	No	No	No	No	No	>1000	43^b^	200	ND	ND	GG	C**G**	A**T**	**CC**	02, 68	35, 44
11	M	PDL/PDL	49	No	No	No	No	No	No	>1000	99	87^b^	235	76	GG	C**G**	A**T**	**CC**	11, 32	40, 44
12	M	PDL/PDL	42	No	No	No	No	No	No	345^b^	76	191	23	67	GG	**GG**	AA	**CC**	30, 68	08, 35
13	M	ND	68	No	No	No	No	No	No	726	54	143^b^	31	60	GG	**GG**	AA	**CC**	02, 33	44, 51
14	M	ND	55	No	No	No	No	No	No	519	35	128	24	41	GG	**GG**	AA	**CC**	03, 32	49, 49
15	F	ND	37	Yes	No	No	No	No	No	>1000	34^b^	107^b^	32	46	GG	**GG**	AA	**CC**	29, 68	35, 44
16	M	ND	63	No	No	No	No	No	No	620	56	144^b^	65	89	GG	C**G**	AA	**CC**	02, 31	44, 50
17	M	ND	59	No	No	No	No	No	No	>2000	90	210	130	386	G**A**	CC	AA	T**C**	02, 03	14, 38
18	M	PDL/PDL^a^	22	No	No	No	No	No	No	511	53	163	64	192	GG	C**G**	AA	T**C**	02, 29	18, 44
19†	M	LAG/LAG^a^	74	No	No	Yes	No	Yes^†^	Yes^†^	>1000	57	136^b^	42	30	GG	C**G**	AA	T**C**	02, 31	13, 40
20	M	PDL/PDL^a^	54	Yes	No	No	No	Yes	No	826	96	173	192	104	GG	C**G**	AA	T**C**	02, 24	50, 57
21	M	PDL/PDL	55	No	No	No	No	No	No	>1000	45^b^	157^b^	51	44	GG	C**G**	AA	T**C**	02, 02	15, 51
22	M	PDL/PDL	42	No	No	No	No	No	No	543	65	166	21	56	GG	C**G**	AA	T**C**	03, 23	49, 57
23	F	PDL/PDL	54	No	No	No	No	No	No	>1000	41^b^	88^b^	34	35	GG	C**G**	AA	T**C**	01, 03	08, 35
24	M	ND	56	No	No	No	No	No	No	447	29^b^	87^b^	21	18	GG	C**G**	AA	T**C**	03, 23	35, 49
25†	M	ND	49	ND	ND	ND	ND	ND	ND	>2000	32^b^	16^b^	63	44	GG	C**G**	AA	T**C**	11, 31	35, 44
26	M	RG/RG	34	ND	ND	ND	ND	ND	ND	2000	44^b^	126^b^	39	39	GG	C**G**	AA	T**C**	03, 68	40, 44
27	F	ND	62	No	No	No	No	No	No	>1000	55	214	1180	1877	GG	C**G**	AA	T**C**	02, 66	39, 40
28	M	RG/RG	51	No	No	No	No	No	No	659	29^b^	132^b^	ND	ND	G**A**	CC	AA	TT	24, 29	35, 37
29	M	ND	56	No	No	No	No	No	No	>1000	42^b^	167	64	75	G**A**	CC	AA	TT	02, 03	07, 07
30	F	PDL/PDL	49	No	No	No	No	No	No	60^c^	52	160	68	164	G**A**	CC	AA	TT	02, 29	18, 37
31	M	LAG/LAG	20	No	No	No	No	No	No	592	46	157^b^	85	175	G**A**	CC	AA	TT	01, 03	35, 51
32	M	NOR/NOR^a^	33	ND	No	No	No	No	No	>1000	62	254	147	159	GG	CC	AA	T**C**	02, 24	14, 35
33	F	NOR/POV	49	Yes	No	No	No	No	No	28^c^	46	218	28	46	GG	CC	AA	T**C**	02, 30	42, 51
34	M	RG/RG^a^	63	No	No	No	No	No	No	827	90	271	41	49	GG	CC	AA	T**C**	01, 24	15, 27
35	M	RG/RG^a^	54	ND	No	No	No	Yes	No	408	89	142^b^	193	68	GG	CC	AA	TT	02, 30	13, 40

wt, wild-type; ND, not determined; TS, transferrin saturation; AST, aspartate transaminase; ALT, alanine transaminase; F, female; M, male;

^a^, the parents were born in the same locality;

^b^, observable values non-concordant with the expected ones;

^c^, values below the expected since the patient menstruates.

### *HFE* mutation analysis

*HFE* mutation analysis − c.187C>G [p.His63Asp], c.193A>T [p.Ser65Cys], c.340+4T>C [IVS2+4T>C], and c.845G>A [p.Cys282Tyr] − was performed by several techniques. The detection of c.187C>G, c.193A>T, and c.845G>A was carried out by two methods: *i*) polymerase chain reaction followed by specific restriction enzyme (PCR-RFLP) [15] for 35 patients and 469 general population individuals, or *ii*) real-time PCR using TaqMan^®^ genotyping assays for 6 patients.

In RFLP analysis, PCR products from exons 2 (208 base pairs, bp, for c.187C>G and c.193A>T) and 4 (390 bp for c.845G>A) were digested for 2 hours at 37°C with *Rsa*I for c.845G>A, *Mbo*I for c.187C>G and *Hinf*I for c.193A>T (New England Biolabs). The digestion products were size resolved by electrophoresis on 4% agarose gel and visualized by SYBR^®^ Green I nucleic acid gel stain (Molecular Probes). For the c.845G>A, the *Rsa*I produced two fragments of 250 and 140 bp in the wild type DNA and three fragments of 250, 111 and 29 bp in the mutated DNA. In the case of wild type DNA for c.187C>G, the *Mbo*I generated two fragments of 138 and 70 bp, whereas for *Hinf*I the two fragments have 147 and 61 bp; both c.187C>G and c.193A>T mutated DNA were not cut.

In the real-time PCR analysis, we genotyped the c.187C>G (rs1799945) and c.845G>A (rs1800562) mutations by TaqMan^®^ Pre-Designed SNP Genotyping Assays on an ABI 7500 Fast Real-Time PCR System, according to the manufacturer’s instructions (Applied Biosystems). For the c.193A>T mutation, an in-house assay was developed [16] as follows: a 25 μl reaction consisting of TaqMan^®^ genotyping PCR master mix, 32 ng of genomic DNA, 300 nM of each primers 5'-TTGGGCTACGTGGATGACC-3' and 5'-TCTGGCTTGAAATTCTACTGGAAA-3' and 150 nM of each TaqMan^®^ MGB probes, 5'-VIC-ACGGCGACACTCANFQ-3' (mutated) and 5'-FAM-CGGCGACTCTCANFQ-3' (wild-type). The PCR conditions were as follows: 1 min at 60°C, 10 min at 95°C, followed by 40 cycles of 15 sec at 95°C and 1 min at 60°C.

Considering patients’ clinical manifestations and their *HFE* status (the majority of patients are not c.845A homozygous), we also performed direct sequencing of *HFE* exons 2, 4 and 5, including exon-intron boundaries. For this, we used the primers described above for RFLP analysis; however, for exon 5 the following primers were used: 5'-GATGAGAGCCAGGAGCTGAG-3' and 5'-CCCTGGGGCAGAGGTACT-3'. The exon 2 and its intron-flanking sequences analysis allows for the identification of the c.340+4T>C [IVS2+4T>C] splice site variation, which has been reported as associated with iron overload [11], whereas sequencing of exon 5 and its intron-flanking regions allows for the evaluation of the splice site mutation c.1008+1G>A [IVS5+1G>A] [13]. Each 20 μl PCR amplification reaction contained 100 ng of genomic DNA, 10 μM primers, 200 μM dNTPs (Promega), 25 nM MgCl_2_ (Qiagen), 1x Q-Solution (Qiagen), 1x buffer (Qiagen), 5 U of HotStart Taq (Qiagen), and sterile water. The PCR started with an enzyme activation step at 95°C for 15 min, then 40 cycles at 94°C for 30s, 56.5°C for 30s and 72°C for 1 min, followed by a final extension step at 72°C for 10 min. Amplification products were purified using the QIAquick PCR Purification kit (Qiagen), according to the manufacturer’s instructions. Purified products were sequenced, using the same primers of the PCR amplification, with the BigDye Terminator v1.1 cycle sequencing kit (Applied Biosystems) under the following conditions: 1 μl ready reaction mix, 5 μl BigDye sequencing buffer, 3.2 pmol of forward or reverse primer, 7 ng DNA, and sterile water to a final reaction volume of 20 μl. Cycle sequencing was performed using a initial denaturation step at 96°C for 1 min followed by 25 cycles at 96°C for 10s, 50°C for 5s, and 60°C for 4 min in a GeneAmp^®^ PCR System 2700 (Applied Biosystems). The sequencing products were purified with a BigDye XTerminator^®^ Purification kit, and separated by capillary electrophoresis in an automated sequencer (ABI 3130 Genetic Analizer, Applied Biosystems) with a 36 cm length capillary and POP-7^TM^ polymer, according to the manufacturer’s instructions. Data were analyzed with the Sequencing Analysis software version 5.3.1 (Applied Biosystems). The alignment and edition of sequences were carried out using the Bioedit™ software version 7.0.0.

Sequencing results revealed the presence, in some patients, of the splice site variation c.340+4T>C (rs2071303). Consequently, this variant was genotyped in the general population (469 individuals) by TaqMan^®^ Pre-Designed SNP Genotyping Assays (Applied Biosystems) on an ABI 7500 Fast Real-Time PCR System, according to manufacturer’s instructions.

### *HLA* genotyping

*HLA*–A and–B genotyping was performed in the 41 patients by PCR amplification with sequence-specific primers (PCR-SSP), according to the manufacturers’ instructions (Olerup SSP™, GenoVision Inc.). After electrophoresis on a 4% agarose gel stained with SYBR^®^ Green, the PCR products were visualized, followed by *HLA* group allele identification using the Helmberg-SCORE^TM^–Sequence Compilation and Rearrangment Evaluation, for research only—software (*Olerup* SSP AB). This methodology only allows a low resolution genotyping. Consequently, *HLA* was characterized in a group level resolution, and the specific alleles present in each group were not discriminated.

### Statistical analysis

Allele and genotype frequencies were estimated for the *HFE*-c.187C>G, -c.193A>T, -c.340+4T>C, and -c.845G>A mutations in the general population (n = 469). Hardy-Weinberg equilibrium (HWE) for the general population was determined by the Arlequin software v.3.5.1.2. No departure from HWE was observed. Then, contingency tables were constructed to calculate the statistical differences between the allele frequencies in the six municipalities of the island. Fisher’s exact test was used instead of a chi-square test, since the last may be inapplicable for very small expected frequencies (number of subjects in a cell should be 5 or more). Data analysis was carried out using the statistical package SPSS software, version 10.0 (SPSS, Inc.). The results were considered statistically significant when the *p* values were less than 0.05.

The *HLA*–A and–B group alleles’ frequencies were calculated by direct counting. The *HFE-*c.845A-*HLA-*A–B haplotypes were directly obtained by segregation analysis through five consecutive generations in the family affected with pseudodominant inheritance of HH, and by–A and–B group alleles’ homozygosity in two unrelated patients (patients 3 and 5). The same strategy was applied to determine the *HFE-*c.187G-*HLA-*A-B (patient 14). For a random subset (93 individuals) of the general population, the *HLA* haplotypes were estimated with the expectation-maximization (EM) algorithm provided in the Arlequin package, as previously described [17].

Association analysis of iron overload with *HFE* and *HLA* haplotypes, as well as with *HFE* genotypic profiles, was performed by calculating relative risks (RRs) with 95% confidence intervals (CI) using the 2-way Contingency Table Analysis webpage (http://statpages.org/ctab2x2.html).

## Results

### Study of a family affected with pseudodominant inheritance of hereditary hemochromatosis type-I

**Fig 1:**
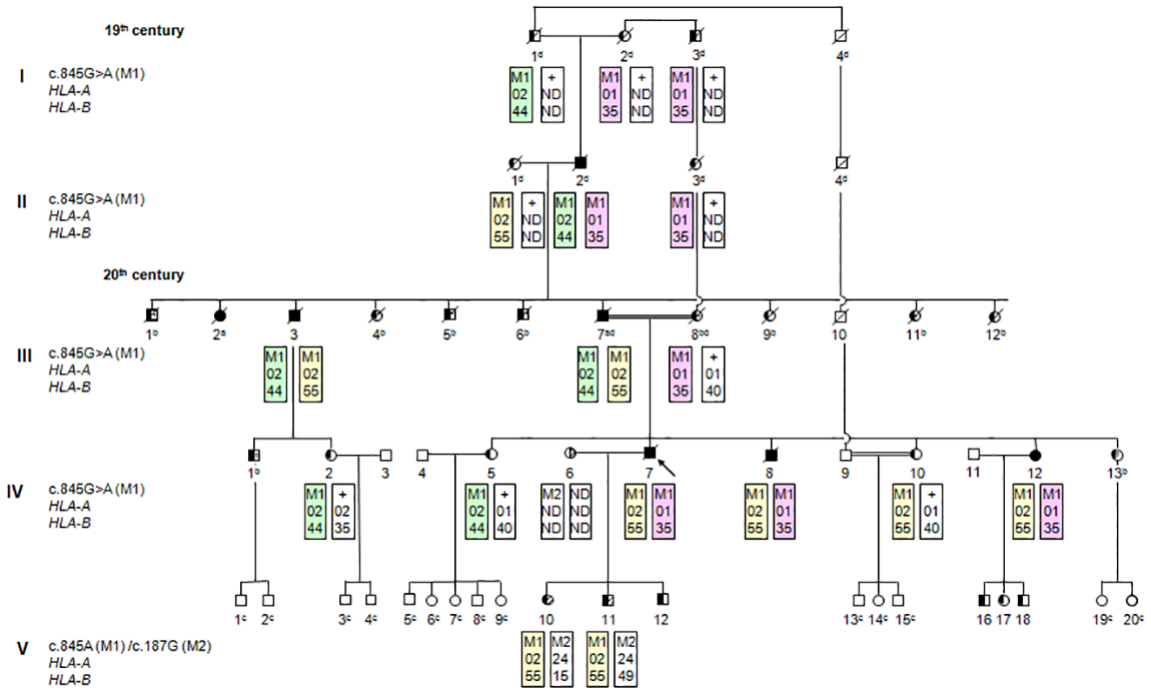
Family from São Miguel Island (Azores) with pseudodominant inheritance of hereditary hemocromatosis type-I caused by the segregation of *HFE* mutated alleles. The segregation of *HLA*-A–B haplotypes associated with *HFE-*c.845A mutation is also presented. “a” designates the homozygous subjects genotyped by clinical and biochemical criteria; “b” indicates the individuals with an unknown allele; “c” are the non determined subjects; and “d” are subjects with inferred genotype.

The study of hereditary hemochromatosis in São Miguel Island began in 1988 by the identification of two brothers—the proband (patient IV.7) and one sibling (patient IV.8)–with a classical clinical picture of HH and one paternal uncle affected with a minor form of the disease (III.3, Fig 1). In 2000, after the introduction of the *HFE* gene testing, this family, which is from a small village with less than 600 inhabitants, was reinvestigated in order to understand the HH segregation and to offer a better follow up of their relatives [18]. Their family pedigree, which spans five generations, is shown on Fig 1. The HH phenotype was identified in nine family members, from II to V consecutive generations: three patients (II.2, III.2 and III.7) by clinical and family history, and six patients (III.3, IV.7, IV.8, IV.12, V.10 and V.11) by clinical, biochemical, and *HFE* genotyping. A summary of the demographic, clinical, biochemical and genetic data of these patients is shown in Table 1. The proband (42y, IV.7) and one brother (40y, IV.8) presented the classical clinical picture of hereditary hemochromatosis type-I, including a transferrin saturation higher than 80% and a serum ferritin higher than 1000 ng/mL. The paternal uncle (74y, III.3) had only a slightly elevated serum ferritin. They were all homozygous for the mutated allele *HFE*-c.845A [p.282Tyr]. Following the characterization of these first three patients, a biochemical and genetic screening was given to 11 additional family members. First, we investigated three sisters (IV.5, IV.10 and IV.12) of the two affected brothers and the daughter (IV.2) of the affected paternal uncle. The mutation analysis revealed that one clinically asymptomatic sister (37y, IV.12) was *HFE*-c.845A homozygous, being the other three heterozygotes for this mutation (c.845GA). We performed a clinical evaluation and genetic testing to the other five family members living in the São Miguel Island, namely the proband spouse (IV.6), three husbands of proband sisters (IV.4, IV.9 and IV.11), and the husband of one first-cousin (IV.3). The *HFE* analysis showed that the spouse of the proband (IV.6), which is non-related, is heterozygous for the c.187G [p.63Asp] mutation. Therefore, the molecular screening was also offered to their three children, aged 20 (V.10, female), 18 (V.11, male) and 15 (V.12, male) years-old, since they were suspected to be *HFE*-c.845A/c.187G compound heterozygous. This hypothesis was confirmed in the daughter (V.10) and in one son (V.11). Both were clinically asymptomatic, but presented high transferrin saturation levels.

### *HFE* mutation analysis of unrelated patients with biochemical evidence of iron overload

*HFE* genotyping for the three most common mutations associated with iron overload was performed in 35 unrelated patients from the São Miguel Island (Azores) who were referred by their physicians due to suspicion of the classical form of hereditary hemochromatosis (type-I), based on biochemical criteria of iron overload (see M&M). A summary of the demographic, clinical, biochemical, and genetic data of these patients is also presented in Table 1. Six of the 35 patients (17%) were homozygous for *HFE*-c.845A [p.282Tyr], and three were c.845A/c.187G compound heterozygous. In these nine patients, the predominant clinical manifestations were skin pigmentation and arthropathy; three patients presented liver cirrhosis, one in association with classical hemochromatosis and bronze diabetes (patient 5 in Table 1). Although the highest TS values were found in patients homozygous for c.845A mutation, there was not, in general, a clear-cut correlation between patients’ genotype and biochemical data.

Sequencing of *HFE* exons 2, 4 and 5 and their intron-flanking regions in all patients (6 familiar and 35 unrelated) did not reveal any other mutation, but allowed the identification of the c.340+4C [IVS2+4C] splice site variant in 26 patients (Table 1), either in a heterozygous (19) or in a homozygous state (7). In three patients (#32–34; Table 1), this variant was the only *HFE* mutation present. Overall, considering the four *HFE* mutations– c.187C>G [p.His63Asp], c.193A>T [p.Ser65Cys], c.340+4T>C [IVS2+4T>C], and c.845G>A [p.Cys282Tyr]–around 60% of unrelated patients (21 out of 35) have at least two mutations, found in-*cis* or in-*trans* position, which may explain the iron overload. In seven of these 21 patients (33.3%), the *HFE-*c.187G and -c.340+4C variants are in*-cis* configuration. This result is corroborated by the *HFE* haplotypes and genotypic profiles analysis, where individuals carrying the c.187G allele also carry -c.340+4C (H_2;_ GP_2-5_ and GP_7;_ Tables 2 and 3), and by the linkage disequilibrium (D’ = 0.826) between the two variants. Finally, carriers of both variants have increased risk of developing iron overload (RR = 2.08 95% CI = 1.40 2.94, p<0.001; Table 2 *HFE*-H_2_), despite neither, by themselves, are associated with this condition (c.187G RR = 1.14, 95% CI 0.72–1.72, *p* = 0.546; and -c.340+4C RR = 0.28, 95% CI 0.10–0.66, *p* = 0.001; Table 2 *HFE*-H_4_).

**Table 2:** *HFE* and *HLA* haplotype frequency and association analysis with iron overload. *HFE* haplotypes were indirectly inferred by Arlequin, whereas *HLA* haplotypes were directly inferred by homozygosity.

	Haplotype									
						Frequency		Association analysis		
*Locus*	ID					Iron overload patients	General population	RR	95% CI	*p*-value
***HFE***		c.187C>G	c.193A>T	c.340+4T>C	c.845G>A					
	**H**_**1**_	C	A	T	A	0.326	0.035	**10.40**	**6.51–16.36**	**<0.001**
	**H**_**2**_	G	A	C	G	0.315	0.152	**2.08**	**1.40–2.94**	**<0.001**
	H_3_	C	T	C	G	0.022	0.016	1.53	0.24–6.73	0.569
	H_4_	C	A	C	G	0.098	0.217	0.28	0.10–0.66	0.001
	H_5_	C	A	T	G	0.239	0.509	0.46	0.29–0.67	<0.001
***HLA*** ^**ª**^		**A**	**B**							
	**H**_**1**_	01	35			0.073	0.005	**13.61**	**1.68–299.82**	**0.001**
	**H**_**2**_	02	55			0.073	0.005	**13.61**	**1.68–299.82**	**0.001**
	H_3_	02	44			0.061	0.066	0.95	0.30–2.78	0.913
	H_4_	24	15			0.049	0.005	9.07	0.98–212.98	0.016
	H_5_	03	27			0.024	0.005	4.54	0.33–126.13	0.173
	H_6_	03	50			0.012	<0.001	‒		
	H_7_	03	49			0.037	<0.001	‒		
	H_8_	32	49			0.012	0.016	0.76	0.03–8.00	0.807

^ª^ H_1_-H_6_ and H_7_-H_8_ were associated with *HFE-*c.845A and *HFE-*c.187G, respectively.

**Table 3:** *HFE* genotypic profile frequency and association analysis with iron overload.

*HFE* genotypic profile									
ID					Frequency		Association analysis		
	c.187C>G	c.193A>T	c.340+4T>C	c.845G>A	Iron overload patients	General population	RR	95% CI	*p*-value
**GP**_**1**_	CC	AA	TT	AA	0.244	0.002	**114.39**	**16.04–2386.15**	**<0.001**
**GP**_**2**_	CG	AA	TC	GA	0.122	0.006	**19.07**	**4.10–98.88**	**<0.001**
**GP**_**3**_	CG	AT	CC	GG	0.049	0.005	**11.44**	**1.17–112.26**	**0.002**
GP_4_	GG	AA	CC	GG	0.098	0.039	2.54	0.74–7.40	0.074
GP_5_	CG	AA	CC	GG	0.024	0.062	0.39	0.02–2.49	0.328
GP_6_	CC	AA	TC	GA	0.024	0.023	1.04	0.05–7.31	0.970
GP_7_	CG	AA	TC	GG	0.244	0.188	1.30	0.67–2.27	0.381
GP_8_	CC	AA	TT	GA	0.098	0.051	1.91	0.57–5.33	0.211
GP_9_	CC	AA	TC	GG	0.073	0.202	0.361	0.09–1.06	0.044
GP_10_	CC	AA	TT	GG	0.024	0.278	0.09	0.01–0.52	<0.001

### Association analysis of *HFE* haplotypes and genotypic profiles with iron overload

In order to assess the association of *HFE* haplotypes (H) and genotypic profiles (GP) with iron overload we calculated relative risk (RR) by comparing the 41 patients against the general population. The haplotypes RR results revealed that H_1_^CATA^ (RR = 10.40, 95% CI = 6.51−16.36, *p*<0.001) and H_2_^GACG^ (RR = 2.08, 95% CI = 1.40−2.94, *p*<0.001) confer susceptibility to iron overload (Table 2). On the other hand, a protective effect (*p*<0.05) was observed for H_5_ (wild-type) carriers. *HFE* genotypic profile data demonstrated a significant positive association of profiles GP_1_^CC AA TT AA^ (RR = 114.39, 95% CI = 16.04−2386.15, *p*<0.001), GP_2_^CG AA TC GA^ (RR = 19.07, 95% CI = 4.10−98.88, *p*<0.001) and GP_3_^CG AT CC GG^ (RR = 11.44, 95% CI = 1.17−112.26, *p* = 0.002) with iron overload (Table 3), whereas GP_10_^CC AA TT GG^ showed a protective effect, as expected.

### Genetic background of *HFE-*c.845A and -c.187G mutations and association analysis of *HLA* haplotypes with iron overload

In order to determine the genetic background of *HFE-*c.845A [p.282Tyr] and -c.187G [p.63Asp] mutations, we assessed the *HLA-A–B* haplotypes in patients homozygous for -c.845A and -c.187G. A total of six *HLA* haplotypes (H_1_ –H_6_; Table 2) associated with *HFE-*c.845A mutation were identified: A*01-B*35 (H_1_), A*02-B*55 (H_2_), A*02-B*44 (H_3_), A*24-B*15 (H_4_), A*03-B*27 (H_5_), and A*03-B*50 (H_6_). Of these, three—H_1_, H_2_ and H_3_—were directly inferred by family segregation analysis (Fig 1). The remaining three haplotypes were directly inferred from two *HFE-*c.845A [p.282Tyr] homozygous unrelated patients. These patients also presented homozygosity for *HLA* group alleles: patient 3 for A*03, providing the H_5_ and H_6_ haplotypes, and patient 5 for both A*24 and B*15 group alleles, generating the H_4_. For the latter patient, the extended *HLA* haplotype also revealed homozygosity for group alleles C*03, DRB1*11, DQA1*05 and DQB1*03. Regarding *HFE-*c.187G [p.63Asp] homozygous patients, two *HLA-A-B* haplotypes were directly inferred: patient 14 for B*49 –A*03-B*49 (H_7_) and A*32-B*49 (H_8_).

General population’s *HLA-A–B* haplotypes were determined, by indirect inference, without *HFE*-c.845A and -c.187G carrier information. In this subset, a total of 84 haplotypes were identified, being A*01-B*08 (0.086), A*02-B*44 (0.066), and A*24-B*08 (0.043) the most frequent (S1 Table). The patients’ direct haplotype inference revealed that, with the exception of A*03-B*50 (H_6_) and A*03-B*49 (H_7_), all other patients’ haplotypes were present in the general population (Table 2).

To investigate the association of *HLA* group alleles and haplotypes with iron overload, we calculated relative risk by comparing the 41 patients against the general population. The data demonstrated a significant positive association of B*35 (RR = 2.65, 95% CI = 1.20−5.87, *p* = 0.007) and B*55 group alleles (RR = 6.80, 95% CI = 1.28−48.45, *p* = 0.006) with iron overload; but, when we removed *HFE-*c.845A [p.282Tyr] carriers, which include homozygous and heterozygous individuals, this association became statistically non-significant. Regarding the haplotype analysis, a total of 47 different haplotypes were indirectly inferred in the patient sample; however, we only report results for patients’ directly inferred *HLA-*A–B haplotypes (Table 2). The data showed a positive association for A*01-B*35 (H1: RR = 13.61, 95% CI = 1.68–299.82, *p* = 0.001) and A*02-B*55 (H2: RR = 13.61, 95% CI = 1.68−299.82, *p* = 0.001). Once more, when *HFE-*c.845A [p.282Tyr] carriers were removed, the statistical significance was lost. These results validate the association of these two *HLA* haplotypes with the *HFE-*c.845A mutation. The remaining four *HLA* haplotypes (H_3-6_) did not present a significant risk. Considering *HLA* haplotypes associated with *HFE-*c.187G [p.63Asp], results did not demonstrate an increased risk for iron overload.

### The *HFE* mutations in São Miguel population and its geographical distribution

Since the São Miguel population lives on an island, we studied the prevalence of the four *HFE* mutations. Table 4 summarizes the results concerning allele and genotype frequencies for the *HFE* mutations in 41 patients and 469 general population individuals. The c.845A [p.282Tyr] mutation frequency in São Miguel population was 0.05, predicting one in 400 individuals could be c.845A homozygous (Table 5) and one in 10 individuals should be heterozygous. The c.187G [p.63Asp] had the second highest allelic frequency (0.204), indicating a c.187G homozygosity and heterozygosity frequencies of one in 24 and one in three individuals of the general population, respectively. Taken together, it is expected 2.04% of c.845A/c.187G compound heterozygous (one out of 49, Table 5) in the São Miguel Island population. The allele frequency of the third mutation studied– c.193T [p.65Cys]–was the lowest observed (0.02), which predicts a heterozygote frequency of one out of 25 subjects in the whole population. Finally, the c.340+4C [IVS2+4C] splice site variation showed the highest allelic frequency (0.401), representing a heterozygote frequency of one in two individuals of the general population. Considering genotype frequencies of the 469 subjects, one (0.002) was c.845A homozygous, 26 (0.055) were c.187G homozygous, and five (0.011) were compound heterozygous for c.845A/c.187G (Table 4). The c.193T mutation was found in 19 subjects: two (0.004) were compound heterozygous for c.845A, and five (0.011) were compound heterozygous for c.187G (Table 4).

**Table 4:** Allele and genotype frequencies of *HFE* mutations in patients with iron overload and in the general population from São Miguel Island.

	Allele			Genotype		
		Frequency			Frequency	
*HFE* mutation	ID	Iron overload patients	General population	ID	Iron overload patients	General population
c.845G>**A** [p.Cys282Tyr]	**A**	0.366	0.050	**AA**	0.244	0.002
	G	0.634	0.950	G**A**	0.244	0.096
				GG	0.512	0.902
c.187C>**G** [p.His63Asp]	**G**	0.317	0.204	**GG**	0.098	0.055
	C	0.683	0.796	C**G**	0.439	0.296
				CC	0.463	0.649
c.193A>**T** [p.Ser65Cys]	**T**	0.024	0.020	**TT**	0	0
	A	0.976	0.980	A**T**	0.049	0.041
				AA	0.951	0.959
c.340+4T>**C** [IVS2+4T>C]	**C**	0.402	0.401	**CC**	0.171	0.177
	T	0.598	0.599	T**C**	0.463	0.448
				TT	0.366	0.375
Compound heterozygotes						
c.845**A**/c.187**G**		–	–		0.061	0.011
c.845**A**/c.193**T**		–	–		0	0.004
c.187**G**/c.193**T**		–	–		0.024	0.011

Note. None of the 35 patients were carriers of the c.1008+1G>A [IVS5+1G>A] variant, therefore we do not present data for patients and general population.

**Table 5:** Expected number of individuals homozygous or compound-heterozygous for the *HFE*-c.845G>A [p.Cys282Tyr] or -c.187C>G [p.His63Asp] in Azores, Madeira, and mainland Portugal.

	Expected *HFE* genotype frequencies		
Portugal	c.845GA/c.845A	c.845A/c.187G	Reference
Azores			
São Miguel Island	1 in 400	1 in 49	Present study
Terceira Island	1 in 2347	1 in 132	27
Madeira Island	1 in 84.333	1 in 154	34
mainland			
North and Centre	1 in 368	1 in 49	28
South	1 in 2268	1 in 143	28

**Fig 2:**
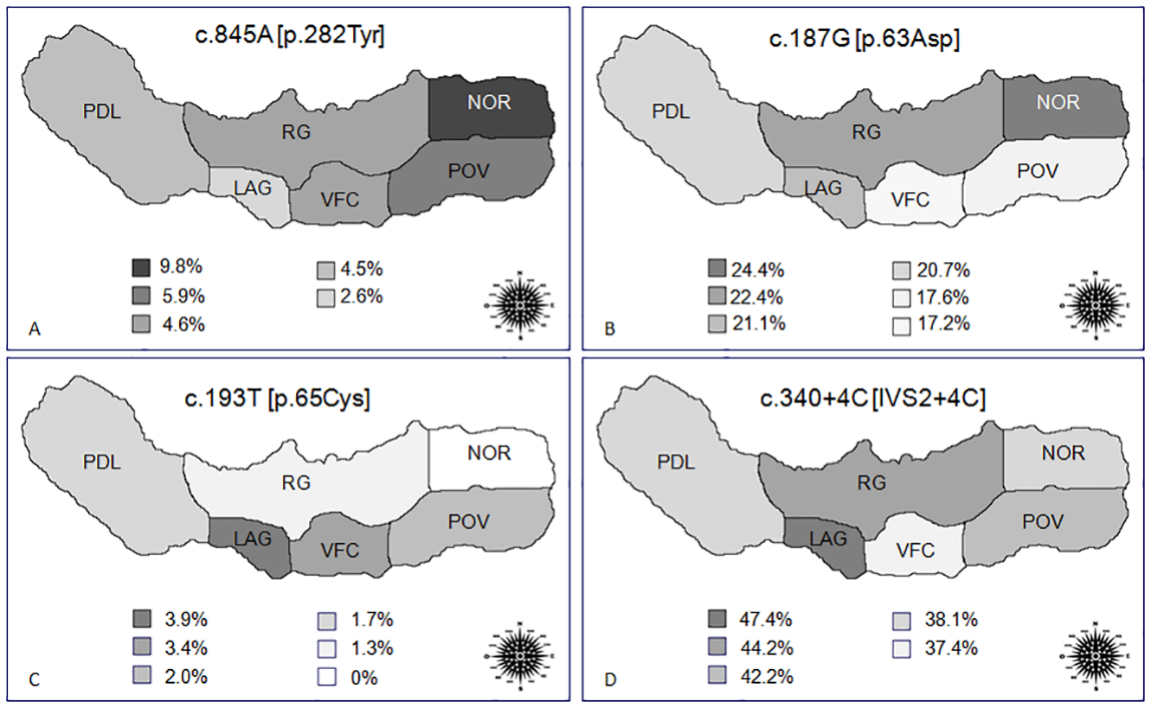
Maps representing the geographical distribution of the allele frequencies (%) of *HFE*-c.845A (A), -c.187G (B), -c.193T (C) and -c.340+4C (D) mutations in the six São Miguel municipalities. PDL—Ponta Delgada; RG—Ribeira Grande, LAG—Lagoa, VFC—Vila Franca do Campo, POV—Povoação, NOR—Nordeste.

In order to assess the relationship between the four *HFE* mutations (c.845A, c.187G, c.193T, and c.340+4C) and their geographical distribution in the São Miguel Island, we compared allele frequencies between the six municipalities (Fig 2). For c.845A, the highest value is found in Nordeste (9.8%) followed by Povoação (5.9%), and the lowest in Lagoa (2.6%). We observed a significant difference (*p* = 0.048) between Nordeste and the other five municipalities (PDL, RG, LAG, VFC, and POV), indicating a relatively non-uniform island distribution for the c.845A mutation. On the other hand, the other three variants– c.187G, c.193T, and c.340+4C –showed a uniform pattern with no significant differences among municipalities.

Considering that the São Miguel population has an admixed genetic background composed mainly of Europeans and less by Jews and Africans [19–25], we compared the *HFE* mutations frequencies with other populations. The analysis revealed that islanders have a c.845A [p.282Tyr] frequency similar to that found in several countries in northern Europe (≈5.7%) [26], but significantly different from the reported frequencies for the Azorean island of Terceira (2.1%) [27]. Regarding c.187G [p.63Asp], the highest frequency observed in Portugal has been detected in the Madeira archipelago (20.5%), followed by São Miguel Island (20.4%), north and center mainland (19.7%) [28] and Terceira Island (18.3%) [27]. The frequencies detected for these two *HFE* mutations (c.845A and c.187G) in São Miguel are significantly higher than those found in Jewish (1.3 and 9.7%) [5] and African (0.9% and 13.2%) [4] populations. As expected, the c.340+4C variation presented similar allelic frequency with phase III CEU HapMap population (34.1%).

## Discussion

Iron overload disorders represent a heterogeneous group of conditions resulting from inherited and acquired means. Despite recent advances, a better understanding of the molecular basis of iron overload is needed in order to improve patient's disease outcome through early diagnosis and treatment. Here, we carried out, for the first time, a clinical evaluation of 41 iron overload patients from the Azorean island of São Miguel (Portugal), which were referred for *HFE* genotyping. Of these, six patients belong to a family with a pseudodominant inheritance of HH. Furthermore, we assessed the frequency of four *HFE* mutations– c.187C>G [p.His63Asp], c.193A>T [p.Ser65Cys], c.340+4T>C [IVS2+4T>C] and c.845G>A [p.Cys282Tyr]–in 469 healthy individuals living on the same island. Association analysis of *HFE* haplotypes and genotypic profiles with iron overload were also evaluated. Finally, we studied the *HLA*-A and -B group alleles and haplotypes linked with the *HFE-*c.845A and *HFE-*c.187G mutations.

In the present research, the patients’ clinical evaluation demonstrate that around 43.9% (18 out 41) present serum ferritin higher than 1000 ng/mL, and 29.3% (12 out 41) show HH’s clinical manifestations, the most frequent being hyperpigmentation and hepatic cirrhosis. Moreover, there is a higher frequency of *HFE*-c.845A homozygous individuals in the patient cohort (24.4%) compared to the general population (0.2%). The majority of patients presenting an iron overload did not fulfill the criteria of HH, suggesting that additional environmental or genetic factors could contribute to iron overload. Sequencing of *HFE* exons 2, 4 and 5, and intron-flanking regions did not reveal any other mutation, but allowed the identification of c.340+4C [IVS2+4C] splice site variant in 63.4% patients (26 out of 41, Table 1). This variant alone does not explain iron overload (RR = 0.28, 95% CI 0.10–0.66, *p* = 0.001; Table 2 *HFE*-H_4_); however, around 7% (3 out 41) of patients (#32–34, Table 1) were heterozygous for c.340+4T>C and did not show any other *HFE* mutation. Bioinformatic analysis using the Human Splicing Finder 2.4 (http://www.umd.be/HSF/) [29] revealed that c.340+4T>C has a splicing Δ consensus of +9.71%, a value higher than the expected 7% for a +4 position of 5’ splice site. In fact, this significant impact on splicing is consistent with the biological evidence of exon 2 skipping observed in a patient with histologically-demonstrated iron overload [11]. According to functional studies performed by Martins et al. [14], the protein produced by the c.340+4T>C alternative splicing is retained in the endoplasmic reticulum and do not efficiently reach the plasma membrane with the β_2_-microglobulin chaperone. A similar situation is observed with the p.282Tyr mutated HFE protein.

Although the pattern of inheritance of HH is usually horizontal, *i*.*e*. all patients belong to the same generation, as expected for an autosomal recessive disease, here we describe a vertical (pseudodominant) pattern due to the segregation within the studied family of at least three *HFE* mutant alleles in each generation. Common causes for a pseudodominant inheritance pattern are: i) birth of an affected child from an affected individual and a genetically related (consanguineous) reproductive partner, who is an unsuspecting carrier, and ii) high carrier frequency, enhancing the risk that the spouse of a patient is a carrier of a mutation in the same gene [30]. Interestingly, these two conditions are observed in their family pedigree: the proband’s (IV.7) parents are consanguineous and responsible for the transmission of the c.845A allele and the proband’s spouse (IV.6), who is genetically unrelated, carries the second most frequent mutation– c.187G.

The knowledge of mutation origin improves the comprehension of population genetic background and evolution. The analysis of co-segregation of *HFE* mutations and *HLA*–A and–B with HH in the family pedigree revealed three non-ancestral *HLA-*A-B haplotypes associated with the *HFE-*c.845A mutation: A*01-B*35, A*02-B*44 and A*02-B*55. The first two were also observed in *HFE-*c.845A homozygous HH patients from the north of Portugal [31]. However, the third haplotype (A*02-B*55) and the A*24-B*15 are, to our knowledge, two new non-ancestral haplotypes associated with this mutation. These two haplotypes reinforce the association of *HFE-*c.845A mutation with A*02 (linked to, for example, B*07, B*14 and B*35) or A*24 (linked to B*18, B*35 and B*57) group alleles, both observed in northern Portuguese *HFE-*c.845A homozygous patients [31]. Regarding *HFE-*c.187G, a previous study [32] reported a significant association of this mutation with A*29 or B*44 group alleles, as well as with A*29-B*44 haplotype in the mainland Portuguese population. The obtained results only show association of the *HFE-*c.187G mutation alleles with A*03-B*49 or A*32-B*49. Nevertheless, patient 9, who is c.845A/c.187G compound heterozygous, also presents the A*29-B*44 haplotype.

In order to investigate the geographical distribution of the *HFE*-c.187C>G, -c.193A>T, -c.340+4T>C, and -c.845G>A mutations in the São Miguel Island, we compared the allele frequencies between the six municipalities (Fig 2). The data demonstrate a higher frequency of c.845A mutation in Nordeste compared to the other five municipalities (*p* = 0.048), suggesting a geographic cline from east to west. This observation may lead to theorize the presence of a founder effect; however, the high diversity of *HLA* haplotypes associated with this mutation does not corroborate this hypothesis. Furthermore, this trend was not observed for the other three *HFE* variants– c.187G, c.193T and c.340+4C –, which show no significant differences among the municipalities. Additionally, we cannot rule out the hypothesis that this result may be due to the small sample size. Nevertheless, the observed pattern validates the importance of carrying out screening studies of recessive mutations in relatively small populations like the Azorean island of São Miguel.

In general, the frequency of c.845A [p.282Tyr] in Europe shows a decreasing north-south cline, with values ranging from 5 to 10% in north Europe, and from 1 to 5% in central and south Europe [33]. The frequency of the c.845A mutation in São Miguel was similar to the one reported in north/central mainland Portugal [28], but significantly higher than in the Azores Terceira Island [27], south mainland Portugal [28], and Madeira Island [34]. This data validates previous results where mainland Portuguese, especially from north/center, were the main contributors to the Azorean settlement. Moreover, these results may be suggestive of different population dynamics between the Azorean islands. Comparison with other populations shows that c.845A frequency is similar to central European countries, reflecting our previous results, where Flemish, French, and Germans also contributed to the settlement of the Azores [21–24].

Concerning the c.187G [p.63Asp] mutation, the highest frequency observed in Portugal has been detected in the Madeira archipelago followed by São Miguel Island. This high frequency is similar to that found in southern Europe. Although the relationship between this mutation and HH is unclear, it constitutes a genetic predisposing factor causing iron overload when present with another genetic (*HFE* or other gene mutation) or an environmental factor [35]. The results indicated that alone c.187G is not associated with iron overload, but together with c.340+4C [IVS2+4C] splice variant is responsible for an increase in the risk of developing the disease. This result is corroborated by simulation studies, where adding just one patient homozygous for the mutated allele of both variants (GP_4_^GG AA CC GG^, Table 3) had a significantly increase in risk (RR = 3.10, 95% CI 1.03–8.24, *p* = 0.016). We hypothesize that double homozygous individuals will produce two altered HFE proteins—the p.63Asp [c.187G] mutated protein, and the protein lacking 88 amino acids encoded by exon 2. Overall, these data point to the complex allele *HFE* c.[187C>G;340+4T>C] being an iron overload genetic predisposition factor.

The c.193T [p.65Cys] mutation, also considered a polymorphism, may, alone or combined with other mutations, be associated with mild iron accumulation [36]. In the present study, the small number of patients carrying the c.193T polymorphism makes it difficult to establish conclusions about the relation of iron overload with this variant. However, genotypic profile analysis revealed that carriers of GP_3_^CG AT CC GG^ present a significant risk (RR = 11.44, 95% CI 1.17–112.26, *p* = 0.002; Table 3). In terms of gene expression, these individuals will most likely produce three types of *HFE* altered proteins: the p.63Asp [c.187G] protein, the p.65Cys [c.193T] protein, and the protein missing exon 2 encoded amino acids.

In summary, we provide evidence that at least the complex allele *HFE*-c.[187C>G;340+4T>C] has a role, as a genetic predisposition factor, on iron overload in the São Miguel population (Azores, Portugal). Independent replication studies in other populations are needed to confirm this association. Additionally, the *HFE-*c.845A [p.282Tyr] mutation has a diverse *HLA* genetic background, since it is associated with six haplotypes, three of them described here for the first time—A*02-B*55, A*24-B*15 and A*03-B*50. These data validate the importance of carrying out epidemiological studies of recessive mutations in relatively small populations like São Miguel Island and are a valuable contribution to a perspective study on iron overload in this population.

## Limitations

The present work has some limitations, the major one being the small number of patients; however, it includes practically all island’s diagnosed cases of iron overload that meet the inclusion criteria and is the first report on *HFE* mutation distribution in the healthy population of São Miguel Island. Nonetheless, replication with a larger sample size is needed in order to validate the results observed here. Another constraint is the lack of functional analysis of the complex allele *HFE* c.[187C>G;340+4T>C].

## Supporting Information

S1 Table HLA-A-B haplotypes observed in the São Miguel Island general population. (DOCX)
