# Supplementary material for: Carriers of the Complex Allele HFE c.[187C>G;340+4T>C] Have Increased Risk of Iron Overload in São Miguel Island Population (Azores, Portugal)
Source: PLoS One. 2015 Oct 26;10(10):e0140228. doi: 10.1371/journal.pone.0140228 (PMC4621060; doi:10.1371/journal.pone.0140228)
Supplement: S1 Table — (DOCX) [file pone.0140228.s001.docx]

**Supplementary Table 1.** HLA-A-B haplotypes observed in the São Miguel Island general population.

| ***HLA* Haplotype** | | | | | | | | |
| --- | --- | --- | --- | --- | --- | --- | --- | --- |
| **ID** | **A*** | **B*** | **Frequency** |  | **ID** | **A*** | **B*** | **Frequency** |
| H1 | 01 | 07 | 0.015 |  | H43 | 24 | 51 | 0.012 |
| H2 | 01 | 08 | 0.086 |  | H44 | 24 | 57 | 0.005 |
| H3 | 01 | 15 | 0.005 |  | H45 | 25 | 18 | 0.005 |
| H4 | 01 | 44 | 0.005 |  | H46 | 26 | 47 | 0.005 |
| H5 | 01 | 51 | 0.020 |  | H47 | 29 | 14 | 0.005 |
| H6 | 01 | 57 | 0.019 |  | H48 | 29 | 15 | 0.011 |
| H7 | 02 | 07 | 0.027 |  | H49 | 29 | 38 | 0.011 |
| H8 | 02 | 08 | 0.006 |  | H50 | 29 | 44 | 0.016 |
| H9 | 02 | 13 | 0.005 |  | H51 | 30 | 07 | 0.005 |
| H10 | 02 | 14 | 0.017 |  | H52 | 30 | 18 | 0.016 |
| H11 | 02 | 15 | 0.016 |  | H53 | 30 | 50 | 0.011 |
| H12 | 02 | 18 | 0.005 |  | H54 | 31 | 40 | 0.005 |
| H13 | 02 | 27 | 0.011 |  | H55 | 31 | 49 | 0.005 |
| H14 | 02 | 35 | 0.030 |  | H56 | 31 | 50 | 0.005 |
| H15 | 02 | 40 | 0.011 |  | H57 | 31 | 51 | 0.011 |
| H16 | 02 | 41 | 0.022 |  | H58 | 32 | 18 | 0.005 |
| H17 | 02 | 44 | 0.066 |  | H59 | 32 | 27 | 0.016 |
| H18 | 02 | 49 | 0.016 |  | H60 | 32 | 35 | 0.005 |
| H19 | 02 | 51 | 0.015 |  | H61 | 32 | 38 | 0.005 |
| H20 | 02 | 55 | 0.005 |  | H62 | 32 | 39 | 0.005 |
| H21 | 02 | 58 | 0.005 |  | H63 | 32 | 49 | 0.016 |
| H22 | 03 | 07 | 0.011 |  | H64 | 32 | 53 | 0.005 |
| H23 | 03 | 14 | 0.012 |  | H65 | 32 | 55 | 0.005 |
| H24 | 03 | 18 | 0.010 |  | H66 | 32 | 57 | 0.005 |
| H25 | 03 | 44 | 0.018 |  | H67 | 33 | 07 | 0.011 |
| H26 | 03 | 45 | 0.005 |  | H68 | 33 | 14 | 0.005 |
| H27 | 03 | 51 | 0.005 |  | H69 | 33 | 37 | 0.005 |
| H28 | 03 | 57 | 0.013 |  | H70 | 33 | 39 | 0.005 |
| H29 | 11 | 27 | 0.011 |  | H71 | 33 | 44 | 0.005 |
| H30 | 11 | 35 | 0.005 |  | H72 | 66 | 53 | 0.005 |
| H31 | 11 | 40 | 0.011 |  | H73 | 68 | 07 | 0.005 |
| H32 | 11 | 44 | 0.011 |  | H74 | 68 | 15 | 0.011 |
| H33 | 23 | 41 | 0.005 |  | H75 | 68 | 27 | 0.005 |
| H34 | 23 | 44 | 0.005 |  | H76 | 68 | 35 | 0.015 |
| H35 | 23 | 49 | 0.011 |  | H77 | 68 | 40 | 0.005 |
| H36 | 24 | 08 | 0.043 |  | H78 | 68 | 49 | 0.005 |
| H37 | 24 | 14 | 0.036 |  | H79 | 68 | 51 | 0.007 |
| H38 | 24 | 15 | 0.005 |  | H80 | 68 | 53 | 0.011 |
| H39 | 24 | 18 | 0.006 |  | H81 | 68 | 58 | 0.005 |
| H40 | 24 | 35 | 0.009 |  | H82 | 68 | 78 | 0.005 |
| H41 | 24 | 44 | 0.017 |  | H83 | 80 | 15 | 0.005 |
| H42 | 24 | 50 | 0.016 |  | H84 | 80 | 44 | 0.005 |
